# Patient-Reported Outcomes for Glabellar Line Improvement and Satisfaction With the RelabotulinumtoxinA Ready-to-Use Liquid Formulation: Data From the Phase 3 READY-1 Trial

**DOI:** 10.1093/asj/sjaf063

**Published:** 2025-04-17

**Authors:** Michael H Gold, Lisa Donofrio, Sachin Shridharani, Amir Moradi, Brian Biesman, Melissa Chiang, Rosalyn George, Kristel Polder, Nowell Solish, Rob Schwarcz, Anna-Karin Berg, Felipe Weinberg, Eva Axén

## Abstract

**Background:**

RelabotulinumtoxinA (relaBoNT-A; Relfydess, Galderma, Uppsala, Sweden) is a novel, ready-to-use liquid form of botulinum toxin A, created using PEARL technology to produce a highly effective, complex-free formulation.

**Objectives:**

This study reports the patient-assessed effectiveness and satisfaction outcomes from the relaBoNT-A Phase 3 READY-1 study.

**Methods:**

Adults with moderate-to-severe glabellar lines were randomized 3:1 to receive relaBoNT-A (50 U) or placebo in a 6-month, double-blind, multicenter study. Patient-reported endpoints at maximum frown included ≥1 grade improvement on the glabellar line subject live assessment (GL-SLA) scale from baseline and Global Aesthetic Improvement Scale (GAIS) score. Satisfaction and well-being investigations used the Facial Lines Treatment Satisfaction Questionnaire (FLTSQ), Natural Expressions Questionnaire, and FACE-Q Psychological Function Questionnaire.

**Results:**

Overall, 233 adults received relaBoNT-A and 74 received placebo. RelaBoNT-A responder rates for ≥1-grade GL-SLA improvement from baseline at Day 7 and Months 1, 3, and 6 were 97.2%, 97.7%, 90.0%, and 71.0%, respectively, vs 18.9%, 26.8%, 27.5%, and 22.4% with placebo (*P* < .001). GAIS responder rates were 74.3% to 98.1% (relaBoNT-A) and 9.0% to 16.2% (placebo). Posttreatment FLTSQ Rasch-transformed scores were higher with relaBoNT-A (≥62.5) than placebo (≤49.8) for the Appearance Module and Treatment Satisfaction Module (relaBoNT-A: ≥83.0; placebo: ≤36.8). RelaBoNT-A-treated patients reported looking natural (≥83.3%) and feeling confident when making facial expressions (≥75.7%). Mean change in FACE-Q well-being score was higher with relaBoNT-A (11.0-13.7) vs placebo (0.6-4.5).

**Conclusions:**

Adults with moderate-to-severe glabellar lines receiving a single relaBoNT-A treatment reported significant improvements in glabellar line severity throughout the 6-month study period. RelaBoNT-A provided natural looking results, high satisfaction, and psychological well-being improvements.

**Level of Evidence: 1 (Therapeutic):**

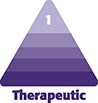

Injections with botulinum toxin A (BoNT-A) are widely used for the nonsurgical aesthetic treatment of age-related lines on the upper face, including lateral canthal lines (crow's feet), horizontal forehead lines, and glabellar lines (frown lines).^1-8^ RelabotulinumtoxinA (relaBoNT-A; Relfydess, Galderma, Uppsala, Sweden) is a novel, complex-free, ready-to-use liquid formulation of BoNT-A, approved in some markets for the treatment of adults (aged >18 years) with moderate-to-severe glabellar lines and lateral canthal lines.^9,10^ RelaBoNT-A is manufactured using PEARL (precipitation-free extraction and activity-preserving refined liquid) technology; a unique proprietary process that yields a high-purity BoNT-A1 product, which retains its natural conformation through filtration and chromatography processes, without precipitation steps.^9-11^ This approach preserves the activity of the core neurotoxin, and the liquid formulation helps to avoid reconstitution-related dosing errors.^9-11^ In addition, relaBoNT-A is free of the animal and human proteins usually found in BoNT-A treatments.^9-11^

RelaBoNT-A has demonstrated efficacy, durability, and a good tolerability and safety profile in the Phase 3 Relabotulinumtoxin Aesthetic Development Study-1 (READY-1) trial; a placebo-controlled, double-blind trial examining treatment outcomes with a single 50 U relaBoNT-A treatment when administered to adults for the correction of moderate-to-severe glabellar lines.^12^ RelaBoNT-A-treated patients demonstrated high composite ≥2-grade responder rates (82.9%) compared with placebo (0%) at Month 1, posttreatment, in patients achieving scores of 0 (none) or 1 (mild) using concomitant investigator- and patient-reported assessments (*P* < .001).^12^ Investigator-assessed response rates for patients demonstrating severity scores of 0 (none) or 1 (mild) remained significantly higher than placebo through Month 6 (*P* < .001), and safety assessments showed a low rate of relaBoNT-A-related treatment emergent adverse events.^12^

Patient-reported outcomes (including satisfaction with treatment) represent a critically important measure of treatment success because aesthetic treatments are primarily sought to improve individuals perception of themselves.^13-15^ Survey data have shown that cosmetically conscious individuals tend to seek aesthetic treatments that offer an established safety profile, and natural-looking results.^16^ Individuals are more likely to request repeat treatments if they are satisfied with the outcomes they observe and have a positive experience regarding treatment tolerability.^13-17^ Satisfaction may also be associated with enhancements in psychological factors (eg, self-esteem) that can influence quality of life (QoL) and overall feelings of well-being.^13-15,18,19^ Indeed, the literature in this area includes many studies in which BoNT-A-treated patients report high rates of satisfaction as well as improvements in feelings of self-confidence and attractiveness and benefits associated with increased QoL following glabellar line correction.^2,4,6,8,13-15,18-20^

Specific tools have been developed to enable standardized approaches to the measurement of satisfaction, well-being, and QoL relating to aesthetic procedures, such as BoNT-A injections.^13-15,21-23^ Satisfaction levels tend to mirror patterns of glabellar line improvements of 1-grade, which emphasizes the value of measuring patient-reported outcomes when evaluating overall treatment efficacy.^2,4,6,8,15,18-20^ The data reported herein examine self-assessed glabellar line severity data, global aesthetic improvement results, patient satisfaction outcomes, and FACE-Q psychological function assessments from the READY-1 study.^12,21-23^ These data aim to provide an understanding of patient-reported treatment effectiveness and satisfaction levels among relaBoNT-A-treated patients as well as insights regarding the impact of treatment on self-assessed aesthetic appearance and psychological well-being.

## METHODS

### Study Design

Methodologies concerning the READY-1 study design, patient inclusion criteria, and treatment (dosing and injection-site pattern) have already been described in detail.^12^ This information is therefore summarized in brief below.

The READY-1 trial comprised a Phase 3, randomized, 6-month, multicenter, placebo-controlled, double-blind study (clinicaltrials.gov, NCT04249583). READY-1 was conducted at 10 centers in the United States and Canada between February 10, 2020 and January 5, 2021. All methodologies complied with the International Council for Harmonization of Technical Requirements for Pharmaceuticals for Human Use Good Clinical Practice and the Declaration of Helsinki. Patients were required to give written informed consent, and ethical approval was obtained from relevant institutional review boards.

Table 1 provides an overview of the different scales used for glabellar line investigator live assessment (GL-ILA) and for patient live assessment of glabellar line severity (GL-SLA), treatment response, satisfaction, and well-being endpoints. The GL-SLA scale is a 4-point categorical scale with text descriptions of wrinkle grades from none to severe for self-reporting of GL severity by patients. The GL-ILA scale is a 4-point scale combining an individual photograph and a descriptive text to support live assessment of wrinkle GL severity by investigators. Both the GL-ILA and GL-SLA scales were validated as part of the clinical development program of Dysport (approved in 2009), and the scales were confirmed appropriate by the FDA for use in clinical development of relaBoNT-A and the subsequent biologics license application. Training on how to use the scales was provided to patients and investigators, respectively.

**Table 1.** Summary of Assessment Scales

GL-ILA photographic scale	GL-SLA static categorical scale	GAIS grading	Facial lines treatment satisfaction/natural expressions questionnaires	FACE-Q psychological function (response scale)
0 (none)	0 (none)	Very much improved	1 (strongly disagree)	1 (definitely disagree)
1 (mild)	1 (mild)	Much improved	2 (disagree)	2 (somewhat disagree)
2 (moderate)	2 (moderate)	Improved	3 (agree)	3 (somewhat agree)
3 (severe)	3 (severe)	No change	4 (strongly agree)	4 (definitely agree)
		Worse		
		Much worse		
		Very much worse		

For FACE-Q assessments, patients indicated the extent to which they agreed with a series of statements relating to psychological well-being. GAIS, Global Aesthetic Improvement Scale; GL-ILA, glabellar line investigator live assessment; GL-SLA, glabellar line subject live assessment.

### Study Population

Adults (aged ≥18 years) with moderate-to-severe glabellar lines (on concomitant GL-ILA and GL-SLA scales) were included in the study. Key exclusion criteria included known allergy/sensitivity to components of the relaBoNT-A formulation or any other botulinum toxin serotype product, previous botulinum toxin facial injections (during the past 9 months) or other relevant facial rejuvenation treatments (within the past 6-12 months), excessive skin laxity, predisposition to/history of eyelid or eyebrow ptosis, current or planned pregnancy, and breastfeeding.

### Study Treatment

Patients were randomized 3:1 at baseline to receive a single treatment on Day 0 with either relaBoNT-A (100 U/mL sterile solution for injection; 50 U total dose) or placebo (buffered sterile solution). The relaBoNT-A product is a ready-to-use solution to be stored at 2 to 8 °C (36-46 °F). Five 0.1 mL injections of relaBoNT-A or placebo were made in the glabellar region at prespecified sites: 1 in the procerus muscle, followed by 2 injection points in the corrugator supercilii muscles on each side, moving outwards from the median. All injections were to be ∼1 cm above the upper orbital rim and internal to the mid-pupillary lines and performed using a 30 to 33 G needle. Posttreatment assessments were conducted at Days 7 and 14 and then monthly at Months 1 through 6. Investigators were authorized to conduct remote study visits through telephone and/or video during the COVID-19 pandemic.

### Study Endpoints

The study protocol specified the primary endpoint (reported by Shridharani et al) of Month 1 composite ≥2-grade responder rate (at maximum frown).^12^ A responder was defined as a patient achieving a score of 0 (none) or 1 (mild) and at least a 2-grade improvement compared with baseline on concurrent GL-ILA and GL-SLA scales.^12^

Patient-reported endpoints for efficacy were assessed at maximum frown during study visits. The GL-SLA responder rate was reported for those patients with ≥1 grade improvement from baseline in severity of glabellar lines. Global Aesthetic Improvement Scale (GAIS) responders were defined as those grading global aesthetic appearance (using a 7-point scale) as “improved,” “much improved,” or “very much improved.”

Patient-reported satisfaction was collected through multiple questionnaires, distributed by the clinic staff, and filled out by the patients on paper. The Facial Lines Treatment Satisfaction Questionnaire (FLTSQ) Appearance and Treatment Satisfaction Modules were used to assess satisfaction at all posttreatment visits. A Rasch-transformed total score, ranging from 0 (worst) to 100 (best), was derived from the sum of each individual's FLTSQ scores. Rasch-transformed scores for the Treatment Satisfaction Module were reported at postbaseline study visits. Rasch-transformed total scores for the Appearance Module were reported at baseline and at each posttreatment study visit. Patients also completed the Natural Expressions Questionnaire at baseline and Months 1 and 6. Responder rate was reported according to the percentage of participants indicating that they “agreed” or “strongly agreed” with statements concerning their appearance during dynamic facial expression (eg, smiling and laughing). Patients completed the FACE-Q Psychological Function Questionnaire at baseline and at Months 1, 3, and 6.^22,23^ A Rasch-transformed total score was calculated using the sum of each patient's FACE-Q Psychological Function scores (according to the FACE-Q manual).^22,23^

### Statistical Analysis

Statistical analyses used version 9.4 of the SAS system, with 2-sided CIs and *P* values calculated at a significance level of 5%.^12^ Patient-reported variables used data from the intention-to-treat (ITT) population, comprising all randomized patients who received relaBoNT-A or placebo injections. Between-group comparisons for ≥1-grade GL-SLA improvement from baseline used the Cochran–Mantel–Haenszel test and were stratified by region (2-sided; 5% significance).

## RESULTS

Overall, 300 patients were randomized and 297 received relaBoNT-A (*n* = 223) or placebo (*n* = 74) in the ITT population (Table 2).^12^ A high proportion of the randomized patients (92%) completed the study until Month 6, with dropout rates of 7% in the relaBoNT-A group and 12% in the placebo group. Most participants were White (83.5%) and 90.2% were female. Mean (standard deviation) age at baseline was 47.6 (12.1) years (range, 21-81 years). Overall, 79.5% had severe glabellar lines (as assessed by investigators using the ILA 4-point photographic scale); 78.5% in the relaBoNT-A arm and 82.4% in the placebo arm. As reported by Shridharani et al, Month 1 composite ≥2-grade responder rate at maximum frown was greater for those treated with relaBoNT-A (82.9%) than placebo (0%; *P* < .001).^12^ The duration of severity improvements, time to return to baseline on both GL-ILA and GL-SLA, was reported to be >24 weeks (as estimated by Kaplan–Meier analysis, based on 212 patients treated with relaBoNT-A and 5 patients treated with placebo, excluding those who did not achieve none-or-mild severity on both GL-ILA and GL-SLA concomitantly).^12^

**Table 2.** Baseline Demographics and Characteristics (ITT Population)

Category	RelaBoNT-A (*N* = 223)	Placebo (*N* = 74)	Total (*N* = 297)
Age at baseline (years)			
Mean (SD)	47.6 (12.41)	47.6 (11.19)	47.6 (12.10)
Minimum, maximum	21, 81	27, 72	21, 81
Sex, *n* (%)			
Female	203 (91.0)	65 (87.8)	268 (90.2)
Male	20 (9.0)	9 (12.2)	29 (9.8)
Race/ethnicity, *n* (%)			
White	186 (83.4)	62 (83.8)	248 (83.5)
Black/African American	17 (7.6)	7 (9.5)	24 (8.1)
Asian	9 (4.0)	2 (2.7)	11 (3.7)
American Indian or Alaska Native	4 (1.8)	0	4 (1.3)
Other	8 (3.6)	4 (5.4)	12 (4.0)
Not Hispanic or Latino	202 (90.6)	64 (86.5)	266 (89.6)
Fitzpatrick skin type, *n* (%)			
I-III	170 (76.2)	51 (68.9)	221 (74.4)
IV-VI	53 (23.8)	23 (31.1)	76 (25.6)
GL-ILA 4-point photographic scale (maximum frown), *n*/*N* (%)			
Moderate	48/223 (21.5)	13/74 (17.6)	61 (20.5)
Severe	175/223 (78.5)	61/74 (82.4)	236 (79.5)
GL-SLA static 4-point categorical scale (maximum frown), *n*/*N* (%)			
Moderate	70/223 (31.4)	27/74 (36.5)	97 (32.7)
Severe	153/223 (68.6)	47/74 (63.5)	200 (67.3)

GL-ILA, glabellar lines investigator live assessment; GL-SLA, glabellar lines subject live assessment; ITT, intention to treat; *N*, number of patients in intention-to-treat population; *n*, number of patients in specific category; RelaBoNT-A, relabotulinumtoxinA; SD, standard deviation.

### Patient-Reported Treatment Effectiveness and Satisfaction Endpoints

Table 3 provides a summary of patient-reported outcomes regarding glabellar line severity improvement (≥1-grade improvement responder rate based on the GL-SLA scale) and GAIS responder rate from baseline as well as Rasch-transformed scores for FLTSQ and FACE-Q assessments. As already reported by Shridharani et al,^12^ the onset of effect was rapid, with 39% of patients reporting in the patient diary that they noticed an effect by Day 1 (1 day after the relaBoNT-A injection), and the median onset of effect by Day 2.

**Table 3.** Summary of GL-SLA ≥1-Grade Improvement, GAIS, FLTSQ, and FACE-Q Assessment Data (ITT Population)

Study visit/ treatment group	GL-SLA ≥1-grade responder rate			GAIS responder rate		FLTSQ Appearance module		FLTSQ Satisfaction module		FACE-Q psychological function appraisal	
	*N*	*n* (%)	*P* value	*N*	*n* (%)	*N*	Mean score	*N*	Mean score	*N*	Mean change from baseline
Day 7											
Placebo	74	14 (18.9)	—	74	9 (12.2)	74	49.8	74	36.8	—	—
RelaBoNT-A	216	210 (97.2)	<.001	217	210 (96.8)	217	68.1	217	86.2	—	—
Day 14											
Placebo	68	20 (29.4)	—	68	11 (16.2)	68	49.5	68	34.1	—	—
RelaBoNT-A	213	207 (97.2)	<.001	213	209 (98.1)	213	74.1	213	88.2	—	—
Month 1											
Placebo	71	19 (26.8)	—	71	10 (14.1)	71	48.5	71	30.1	71	0.6
RelaBoNT-A	218	213 (97.7)	<.001	218	213 (97.7)	218	76.9	218	90.7	218	13.5
Month 2											
Placebo	69	18 (26.1)	—	69	7 (10.1)	69	49.4	69	28.2	—	—
RelaBoNT-A	214	208 (97.2)	<.001	214	207 (96.7)	214	74.5	214	88.6	—	—
Month 3											
Placebo	69	19 (27.5)	—	69	8 (11.6)	69	46.0	69	28.1	69	4.5
RelaBoNT-A	210	189 (90.0)	<.001	210	193 (91.9)	211	71.4	211	86.0	210	13.7
Month 4											
Placebo	68	20 (29.4)	—	68	7 (10.3)	68	47.4	68	27.8	—	—
RelaBoNT-A	210	171 (81.4)	<.001	211	181 (85.8)	210	66.4	209	83.1	—	—
Month 5											
Placebo	67	18 (26.9)	—	67	6 (9.0)	67	45.8	67	25.8	—	—
RelaBoNT-A	208	165 (79.3)	<.001	208	163 (78.4)	208	64.9	208	83.0	—	—
Month 6											
Placebo	67	15 (22.4)	—	67	6 (9.0)	67	45.4	67	28.8	67	1.5
RelaBoNT-A	210	149 (71.0)	<.001	210	156 (74.3)	210	62.5	210	83.3	210	11.0

The sum of each patient's FLTSQ and FACE-Q scores was converted to a Rasch-transformed total score, with a higher total score indicating greater patient satisfaction. For FACE-Q scores, the mean change from baseline is reported. FLTSQ, Facial Lines Treatment Satisfaction Questionnaire; GAIS, Global Aesthetic Improvement Scale; GL-SLA, glabellar line subject live assessment; ITT, intention to treat; *N*, number of patients with an assessment at the study visit and baseline; *n*, number of patients who met the criteria; RelaBoNT-A, relabotulinumtoxinA.

**Figure 1.**
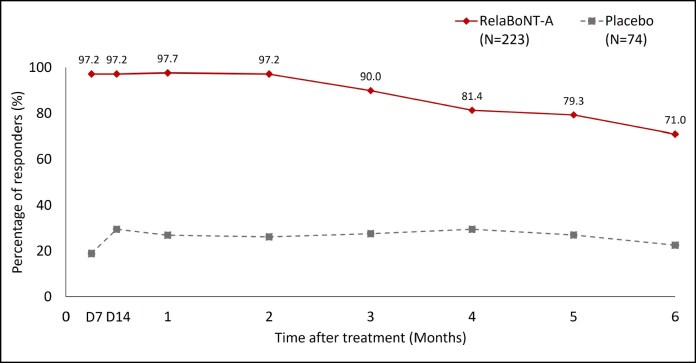
Patient-reported responder rate on the GL-SLA static 4-point categorical scale over the 6-month study period (ITT population). A responder was defined as a patient achieving ≥1 grade improvement from baseline in glabellar line severity score on the GL-SLA scale at maximum frown. *P* value for the between-group difference at each study visit: *P* < .001. Posttreatment study visits and assessments were conducted on Day 7, Day 14, and Months 1 to 6. D, day; GL-SLA, glabellar lines subject live assessment; ITT, intention to treat; RelaBoNT-A, relabotulinumtoxinA.

Responder rates among patients reporting improvements on the GL-SLA scale of ≥1-grade from baseline were significantly higher in the relaBoNT-A arm compared with the placebo arm at all study posttreatment visits (*P <* .001; Figure 1). RelaBoNT-A-treated patients reported responder rates of 97.2%, 97.7%, 90.0%, and 71.0% at Day 7 and Months 1, 3, and 6, respectively. In comparison, responder rates in the placebo group were 18.9%, 26.8%, 27.5%, and 22.4% at Day 7 and Months 1, 3, and 6, respectively.

**Figure 2.**
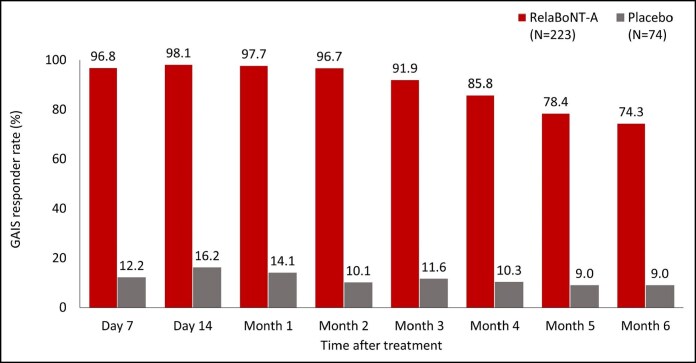
Patient-reported GAIS responder rate at maximum frown during the 6-month study period (ITT population). A responder was defined as a patient who responded as “improved,” “much improved,” or “very much improved” on the patient GAIS at maximum frown. Posttreatment study visits and assessments were conducted on Day 7, Day 14, and Months 1 to 6. GAIS, Global Aesthetic Improvement Scale; ITT, intention to treat; RelaBoNT-A, relabotulinumtoxinA.

Patient-reported GAIS responder rates were high (96.8%) from Day 7, following relaBoNT-A treatment, and maintained at Month 1 (97.7%), Month 3 (91.9%), and Month 6 (74.3%; Figure 2). GAIS responder rates in the placebo arm at the same respective study visits were 12.2%, 14.1%, 11.6%, and 9.0%.

**Figure 3.**
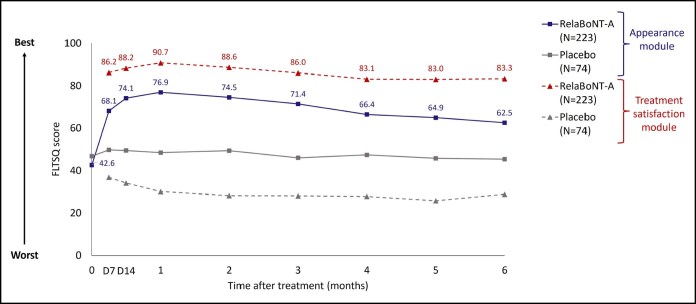
Patient-reported FLTSQ score for the Appearance Module and Treatment Satisfaction Module at maximum frown during the 6-month study period (ITT population). The FLTSQ was used to assess treatment outcome from the patient's perspective. The sum of each patient's FLTSQ scores was converted to a Rasch-transformed total score, with a higher total score indicating greater patient satisfaction. Posttreatment, study visits, and assessments were conducted on Day 7, Day 14, and Months 1 to 6. FLTSQ, Facial Lines Treatment Satisfaction Questionnaire; ITT, intention to treat; RelaBoNT-A, relabotulinumtoxinA.

**Figure 4.**
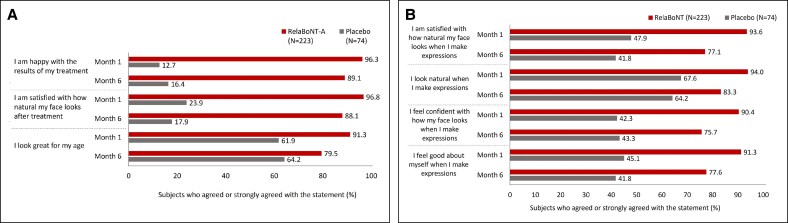
Patient-reported data at Months 1 and 6 after treatment from (A) the FLTSQ (B) the Natural Expressions Questionnaire at Months 1 and 6 after treatment (ITT population). FLTSQ, Facial Lines Treatment Satisfaction Questionnaire; ITT, intention to treat; RelaBoNT-A, relabotulinumtoxinA.

Concerning measures of patient satisfaction, FLTSQ Appearance Module Rasch-transformed total scores were 76.9, 71.4, and 62.5 for relaBoNT-A-treated patients at Months 1, 3, and 6, respectively, and 48.5, 46.0, and 45.4 for patients given placebo (Figure 3). Following relaBoNT-A treatment, FLTSQ Treatment Satisfaction Module Rasch-transformed total scores were 90.7, 86.0, and 83.3 at Months 1, 3, and 6, respectively, whereas placebo group scores were 30.1, 28.1, and 28.8. FLTSQ overall treatment satisfaction remained high at Month 6 with relaBoNT-A (77.1%) compared with placebo (10.4%). Patient-reported FLTSQ data showed that most relaBoNT-A-treated patients were happy with the results of their treatment at Month 1 (96.3%) and Month 6 (89.1%; Figure 4A). The majority of relaBoNT-A recipients felt satisfied with how natural their face looked at Month 1 (96.8%), and this was sustained at Month 6 (88.1%). Patients typically responded that they looked great for their age after relaBoNT-A treatment at Month 1 (91.3%) and most continued to feel the same at Month 6 (79.5%). Natural Expression Questionnaire data revealed that 93.6% and 77.1% of relaBoNT-A recipients felt satisfied with how natural their face looked when they made expressions at Months 1 and 6, respectively (Figure 4B). RelaBoNT-A-treated patients also indicated that they looked natural when making expressions at Month 1 (94.0%) and Month 6 (83.3%). Most relaBoNT-A-treated individuals said that they felt confident (Month 1: 90.4%; Month 6: 75.7%) and good about themselves (Month 1: 91.3%; Month 6: 77.6%) when making facial expressions.

**Figure 5.**
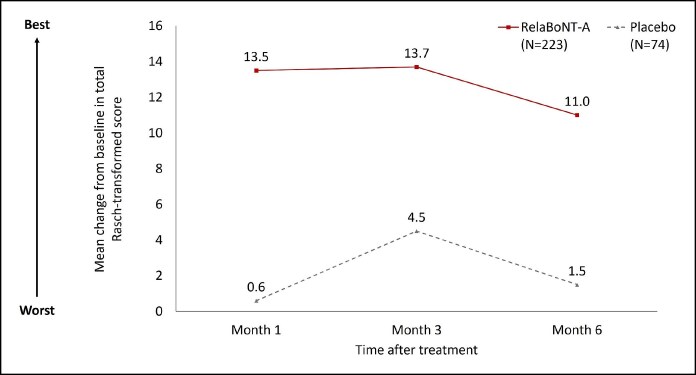
Mean change from baseline in Rasch-transformed score for FACE-Q psychological well-being at Months 1, 3, and 6 after treatment (ITT population). FACE-Q scores were converted to a Rasch-transformed total score, with a higher total score indicating greater patient satisfaction. The mean change from baseline is reported at Months 1, 3, and 6. ITT, intention to treat; RelaBoNT-A, relabotulinumtoxinA.

FACE-Q Psychological Function data showed that patient-reported psychological well-being was higher in the relaBoNT-A arm at Months 1, 3, and 6 compared with placebo (Figure 5). The mean increase in FACE-Q Rasch-transformed from baseline with relaBoNT-A treatment was 13.5 (Month 1), 13.7 (Month 3), and 11.0 (Month 6). In contrast, the increase in Rasch-transformed score was between 0.6 and 4.5 in the placebo arm during the 6-month study period.

**Figure 6.**
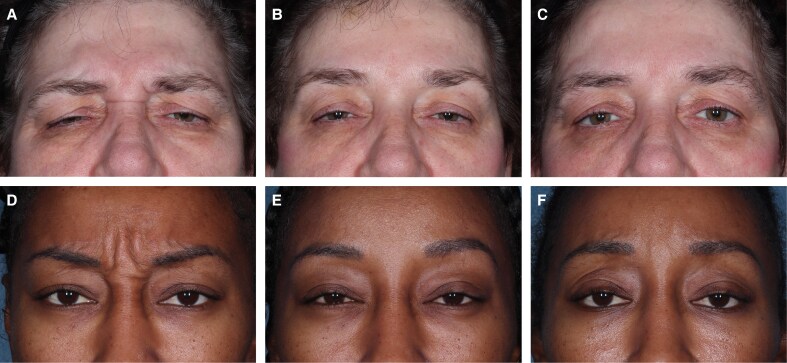
Treatment results in 2 patients injected with relaBoNT-A 50 U in the GLs. Photographs were taken before treatment on Day 0 (A, D), at 1 month (B, E), and 6 months (C, F) after treatment. GAIS was assessed by the patients themselves at maximum frown. (A-C) A 57-year-old woman with severe GLs at maximum frown at baseline (GL-ILA). GAIS results much improved at Months 1 and 6. Very satisfied with treatment at Months 1 and 6. (D-F) A 35-year-old woman with severe GLs at maximum frown at baseline (GL-ILA), GAIS results at Month 1: very much improved; Month 6: much improved; satisfied with treatment at Months 1 and 6.

Photographs of patients before and after treatment with relaBoNT-A are shown in Figure 6. Both of these patients had a 1-grade or greater improvement in GL-ILA and were satisfied or very satisfied with their treatment at Month 6.

### Safety

Safety results from this study have already been published by Shridharani et al^12^ and show that relaBoNT-A injections of GLs were well tolerated, with 3.6% of patients reporting treatment-related adverse events following relaBoNT-A treatment vs 0% in the placebo group, <1% of patients had eyelid ptosis, and no treatment-related pain was reported.

## DISCUSSION

Patient-reported data from the READY-1 study showed that a single 50 U relaBoNT-A treatment resulted in significant reductions in the severity of glabellar lines for adults with moderate-to-severe glabellar lines at baseline, compared with placebo, and most (≥71%) maintained improvements throughout the full 6-month observation period. Patient-reported aesthetic improvement data followed a similar pattern of high and sustained treatment response to that of the previously reported primary efficacy and investigator-assessed outcomes from the READY-1 trial, providing further supporting evidence concerning the scale and durability of relaBoNT-A treatment effect.^12^ Satisfaction with relaBoNT-A was also rated highly throughout the study and accompanied by enhancements in psychological well-being.

Patient-reported data provide support for a rapid onset of effect after relaBoNT-A treatment as well as a long duration of the effects in GLs. In the patient diaries, an effect of treatment was noted as early as 1 day after relaBoNT-A injection in 39% of patients,^12^ and responder rates for GL-SLA ≥1-grade improvements were significantly higher than placebo from Day 7 (*P* < .001) through Month 6. GAIS responder rate with relaBoNT-A treatment was high from Day 7 (96.8%) and maintained by most (74.3%) through Month 6, mirroring the severity improvements and indicating that most patients still regarded their appearance to be improved from baseline at the end of the study period. These treatment outcomes demonstrated in a cohort presenting principally with severe pretreatment glabellar lines mirror published investigator-assessed data from the same study, and broaden the evidence base supporting the efficacy and longevity of glabellar line correction with relaBoNT-A.^12^ Treatment durability may have been because of the high potency of this ready-to-use formulation.^9,10,12^

Satisfaction with aesthetic outcomes has become a widely accepted indicator of BoNT-A treatment success, which may be linked with psychological factors, such as self-confidence, feelings of attractiveness, overall well-being, and QoL.^13-15^ As seen in previous BoNT-A studies, the READY-1 study reported improvements in glabellar line severity of ≥1-grade that were reflective of overall patient satisfaction with relaBoNT-A, which was high from Day 7 and Month 1 and stayed well above baseline for the duration of the 6-month study period.^2,4,6,8,15,19,20^ Most importantly, multiple measures of patient satisfaction consistently showed elevated satisfaction after treatment. FLTSQ scores demonstrated that high satisfaction rates were achieved regarding both appearance and treatment in the relaBoNT-A group from Day 7 and continued to be elevated throughout the 6-month study period. Most relaBoNT-A recipients were happy with their treatment results (≥89%), were satisfied with how natural they looked (≥88%), and reported looking great for their age (≥80%) through Month 6. Previous studies have shown that fear of unnatural-looking aesthetic outcomes represents an important concern among BoNT-A-treated patients, which can influence overall satisfaction with treatment results.^16^ Natural Expressions Questionnaire data from the READY-1 study revealed that relaBoNT-A-treated patients maintained a natural appearance (≥88.6%) and felt satisfied (≥77.1%) and more confident (≥75.7%) during dynamic facial movements through Month 6 compared with placebo-treated patients.

The efficacy and satisfaction outcomes reported during the READY-1 study were accompanied by enhancements in patient-reported measures of psychological well-being, with Rasch-transformed scores remaining improved over baseline (≥11.0) from Months 1 through 6. The FACE-Q Psychological Function questionnaire examines aspects of appearance relating to well-being, including whether the individual feels positive/good about themselves and their level of confidence and attractiveness.^22,23^ These data reveal improvements in important factors relating to the relaBoNT-A treatment experience and provide a broader view of the impact that this new formulation may have on patients, beyond the efficacy, safety, and tolerability results that have already been reported for the READY-1 trial.^12^

With regard to limitations, although several questionnaires and scales were used to investigate multiple aspects of patient satisfaction, this study did not cover the wider impact of GL treatment on, for example, QoL or social function, which would be interesting to study further. Other potential assessments of interest include objective image analysis of aesthetic improvements, which could also complement the patient-reported outcomes presented here. As is commonly observed in aesthetics studies,^24^ there was some placebo-effect in the GL-SLA assessment, which could merit further investigation. However, this likely reflects the high overall patient satisfaction with treatment, because there was less placebo-effect in the investigator assessments in the present study (as previously published).^12^ In addition, in the GAIS and patient satisfaction scales that we report here, the placebo-effect was less evident, suggesting that, taken together, relaBoNT-A treatment of GLs results in consistent improvement in appearance and high patient satisfaction. Further studies examining relaBoNT-A treatment efficacy beyond the 6-month posttreatment time point are warranted to understand the length of treatment effect that may be expected in practice with this innovative BoNT-A formulation.

## CONCLUSIONS

Adults with moderate-to-severe glabellar lines reported statistically significant and durable improvements in the severity of glabellar lines with a single relaBoNT-A treatment. RelaBoNT-A-treated patients reported aesthetic enhancements that looked natural and supported greater confidence in facial appearance, with glabellar line correction being accompanied by high satisfaction levels and improvements in psychological well-being indicators.
